# The Prospective Influence of Trait Alexithymia on Intrusive Memories: What Is the Role of Emotional Recognition Memory?

**DOI:** 10.3389/fpsyg.2018.02642

**Published:** 2019-01-08

**Authors:** M. Roxanne Sopp, Alexandra H. Brueckner, Tanja Michael

**Affiliations:** Division of Clinical Psychology and Psychotherapy, Department of Psychology, Saarland University, Saarbrücken, Germany

**Keywords:** posttraumatic stress disorder, episodic memory, trauma film, intrusions, intrusive re-experiencing, Toronto Alexithymia Scale, dissociation, PTSD

## Abstract

Posttraumatic stress disorder (PTSD) is often considered to be a disorder of memory as patients suffer from fragmented uncontrollable memories (intrusions) whilst experiencing difficulties in intentionally retrieving details of the traumatic event. Recent research suggests that trait-related deficits in the identification of emotional states (alexithymia) may impact emotional memory processes in a way that promotes intrusion formation in PTSD. Therefore, we investigated the influence of alexithymia on intrusive re-experiencing and emotional recognition memory in a prospective analog study. Twenty-six healthy participants took part in a laboratory experiment, which combined two independent paradigms. Participants were exposed to a traumatic film (first session) and completed an episodic memory task comprising neutral and emotional stimuli (second session). In between sessions, participants recorded intrusive memories of the film. Individuals with higher trait alexithymia (HTA) reported an increased number of intrusions on the day of film presentation. Moreover, analyses of memory performance revealed a negative correlation between alexithymia and emotional recognition memory. Further analyses suggest that reduced emotional recognition memory, as evident in individuals with HTA, may, in turn, be associated with enhanced intrusive re-experiencing. As such, the current findings provide first indications regarding the role of alexithymia in emotional learning and PTSD. Future studies should further investigate these associations as well as potential implications for the treatment of PTSD.

## Introduction

Alexithymia refers to a personality construct that comprises several aspects of deficient emotion processing (i.e., difficulties identifying and describing feelings, as well as an externally oriented thinking style; Larsen et al., 2003). These aspects of deficient emotion processing vary considerably between individuals but remain stable across time (Bagby et al., 1994; Martinez-Sanchez et al., 1998; Larsson et al., 2010). Based on this temporal stability, alexithymia is assumed to constitute a trait factor predisposing affected individuals toward a wide array of psychopathological symptoms and conditions (Taylor and Bagby, 2004). Cross-sectional as well as longitudinal findings highlight a particularly strong association between alexithymia and posttraumatic stress disorder (PTSD; Zeitlin et al., 1993; Söndergaard and Theorell, 2004; McCaslin et al., 2006; Frewen et al., 2008a).

PTSD is a mental disorder which may develop after exposure to a traumatic event (e.g., threatened death, injury or sexual assault; American Psychiatric Association [APA], 2013). In the aftermath of a traumatic event, individuals commonly experience stress-related symptoms such as involuntary intrusive memories, hypervigilance, and avoidance of trauma-related stimuli. Although these symptoms commonly remit over time, they persist in approximately 20 to 30% of trauma survivors who go on to develop chronic PTSD (Santiago et al., 2013). As patients with PTSD demonstrate higher alexithymia levels than trauma-exposed healthy controls (Yehuda et al., 1997; Kupchik et al., 2007; Evren et al., 2010; Reeves et al., 2012), it has been proposed that trait alexithymia may influence posttraumatic symptom trajectories (O’Brien et al., 2008). However, evidence in this regard is not unequivocal. Indeed, some accounts suggest that correlations between trait alexithymia and symptom levels may be based on a conceptual overlap between the alexithymia construct and PTSD-specific symptoms of emotional numbing (Badura, 2003; Declercq et al., 2010; Eichhorn et al., 2014). Moreover, the processes that may underlie the association between alexithymia and PTSD symptoms remain largely unexplored. Some studies suggest that secondary effects of alexithymia on emotion regulation and coping strategies may account for the association between trait alexithymia and PTSD development (Boden et al., 2012; Gaher et al., 2016). However, recent findings raise the possibility that alexithymia may directly influence PTSD development via associated deficits in emotional memory processing (Frewen et al., 2008b; Jelinek et al., 2010; Minshew and D’Andrea, 2015).

The latter hypothesis is based on alexithymia’s strong impact on general capacities of affective processing (Taylor and Bagby, 2004). This impact is reflected in neurophysiological responses during emotion processing (van der Velde et al., 2013): Upon presentation of emotional material, high alexithymic individuals show reduced activation changes in the amygdala and in the dorsomedial prefrontal cortex. Moreover, their neurophysiological responses indicate increased cognitive demands during processing of negative and positive stimuli (van der Velde et al., 2013). Alterations in affective processing are also evident in psychophysiological responses to emotional material (e.g., skin conductance and heart rate; Friedlander et al., 1997; Roedema and Simons, 2001; Hua et al., 2014; Kleiman et al., 2016) and have been found to influence attentional processes (Donges and Suslow, 2017). Importantly, there are further indications that trait alexithymia may also affect long-term memory formation. Several studies demonstrate deficits of high alexithymic individuals in episodic memory tests (Taylor and Bagby, 2004; Luminet et al., 2006; Vermeulen and Luminet, 2009; Meltzer and Nielson, 2010; Vermeulen et al., 2010; Donges and Suslow, 2015; but see Nielson and Meltzer, 2009). These deficits are found selectively for emotional material and particularly for negative emotional stimuli (Luminet et al., 2006; Meltzer and Nielson, 2010; Vermeulen et al., 2010; Donges and Suslow, 2015). A recent account by Dressaire et al. (2015) extends these findings by demonstrating that alexithymia is not only associated with impaired memory recall but also with attenuated memory control processes. In a directed forgetting procedure, trait alexithymia was associated with reduced voluntary recall of emotional words, whereas involuntary recall of emotional material was enhanced with increasing levels of alexithymia. These characteristics are strikingly parallel to those found in PTSD patients. It is a common observation in PTSD that patients have limited access to event details of the trauma under intentional retrieval conditions (Halligan et al., 2003; Samuelson, 2011) whilst experiencing recurring intrusive recollections of the traumatic event (Ehlers et al., 2004). These sudden episodes of intense re-experiencing strongly influence the course of symptom development, as they perpetuate continuous distress and perceptions of ongoing threat in PTSD patients (Michael et al., 2005b; Birrer et al., 2007).

Given that the occurrence of re-experiencing symptoms has been linked to peri- and posttraumatic alterations of basic memory processes (Ehlers et al., 2004; Brewin et al., 2010), one could hypothesize that trait alexithymia – via its effects on basic memory processing – may promote intrusive re-experiencing (Bennett and Brooke, 1999). Although a direct test of this hypothesis is currently lacking, preliminary studies have yielded insights into the effects of alexithymia on explicit and implicit trauma memory. Jelinek et al. (2010) examined associations between alexithymia and the emotional word count of explicit trauma memories. They found a negative correlation between trait alexithymia and the emotional word count of trauma narratives in PTSD patients, whereas this association was not present in a trauma-resilient group (but see Peace et al., 2008 for different findings). In a further study, Frewen et al. (2008b) found neurophysiological indications that high alexithymic PTSD patients experience a loss of executive control during script-driven imagery of traumatic events. This loss of executive control may also occur when patients are confronted with reminders of past traumatic events and could thus accelerate re-experiencing symptoms. Finally, Minshew and D’Andrea (2015) investigated implicit and explicit memory for traumatic and threat-related words in survivors of chronic interpersonal violence. Explicit recall of threat-related words was negatively correlated with trait alexithymia, whereas implicit memory for traumatic words was positively correlated with trait alexithymia. This finding is particularly noteworthy as implicit memory for trauma-related material is closely linked to the occurrence of re-experiencing symptoms (Michael et al., 2005a; Michael and Ehlers, 2007).

Despite these promising findings, systematic attempts to investigate the impact of trait alexithymia on intrusive re-experiencing have thus far not been made. A particularly suited approach to investigate influential factors of intrusive re-experiencing in a controlled experimental setting is the trauma film paradigm (Holmes and Bourne, 2008; James et al., 2016). The paradigm exposes healthy participants to a compilation of film scenes displaying extreme physical and sexual violence with the aim of inducing analog PTSD symptoms. After watching the film scenes, participants are typically asked to record intrusive memories of the film as well as associated distress levels during the following days. Using this paradigm, previous studies (Holmes et al., 2004; Laposa and Alden, 2008; Holz et al., 2017) were able to identify several predictors of intrusive re-experiencing (e.g., peritraumatic stress responses, dissociation, and rumination). The predictive value of these factors mirrors cross-sectional associations in clinical samples and aligns with cognitive models of PTSD (Ehlers et al., 2004; Brewin et al., 2010). Hence, the trauma film paradigm can be considered a valid and useful tool for studying the development of re-experiencing symptoms. Expanding on this line of research, the current study investigated the prospective influence of alexithymia on intrusive memories of a traumatic film. In addition, we explored whether deficits in emotional memory performance are linked to trait alexithymia and intrusive re-experiencing. Participants with higher trait alexithymia (HTA) were hypothesized to experience more intrusive memories than participants with lower trait alexithymia (LTA). As intrusive re-experiencing is known to perpetuate general distress and hyperarousal symptoms, HTA participants were expected to report elevated daily stress and arousal levels. Based on previous findings, HTA participants were further hypothesized to show deficits in emotional memory performance. Critically, we tested whether these deficits are linked to enhanced intrusive re-experiencing in HTA participants.

## Materials and Methods

### Participants

Twenty-seven healthy university students took part in the present experiment. The sample was recruited at Saarland University via notices inviting all students aged between 18 and 30 years to participate in a prescreening phase. Thus, as usually the case in experimental studies, a self-selection bias in sampling cannot be precluded. In order to qualify for the study, potential participants were asked to complete an online survey (pretest questionnaire). The questionnaire included the German adaptation of the Toronto Alexithymia Scale (TAS-26; Kupfer et al., 2001) and a mental health questionnaire (Spitzer et al., 1999). Exclusion criteria were acute or chronic somatic illnesses and/or current long-term medication. Potential participants who did not meet these preliminary criteria were additionally required to undergo a semi-structured telephone screening procedure during which previous traumatic experiences, axis I disorders, and previous psychotherapeutic treatment were assessed. If participants had elevated scores on the mental health questionnaire, symptoms of the respective disorder were explored further based on DSM-5 criteria (American Psychiatric Association [APA], 2013). Participants were excluded if any of the following applied: Current mental disorder, previous trauma exposure, insufficient German language skills, pronounced preference for splatter or horror movies, and/or previous familiarization with study materials (traumatic film clips and images from the International Affective Picture System; IAPS; Lang et al., 2005). One of the 27 participants chose to terminate the experiment during exposure to the trauma film and was consequently discarded from further analyses. Thus, the final sample consisted of 26 participants (4 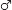 and 22 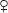) with a mean age of 21.85 years (*SD* = 3.46). The study protocol was approved by the local ethics committee of the Faculty of Human and Business Sciences of Saarland University (Decision n° 15-3). All subjects gave written informed consent in accordance with the Declaration of Helsinki and were paid 25€ for complete study participation.

### Procedure

**FIGURE 1:**
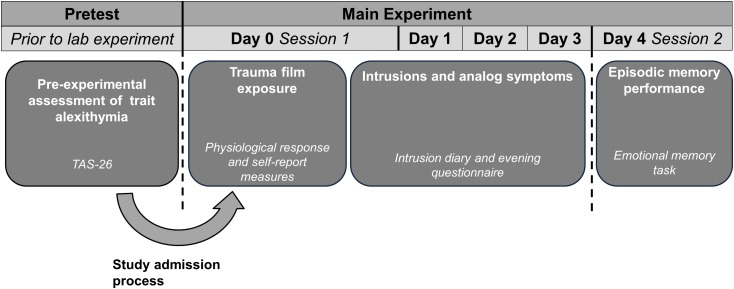
Schematic illustration of the main variables, which were assesses at pretest and on days 0 to 4.

Upon study admission, two individual laboratory appointments were scheduled which were spaced 4 days apart and always took place in the afternoon (01:00 to 04:00 p.m.). The first experimental session consisted of trauma film presentation, including the assessment of subjective and physiological stress measures. Thereafter, participants recorded intrusive memories and daily stress and arousal levels for three consecutive days (see Figure 1). During the second session participants completed the episodic memory task, which included an encoding and a retrieval phase. Episodic memory performance was assessed after completion of the trauma film procedure, as it was necessary to preclude that participants experienced intrusive memories of the task’s stimulus material during the ambulatory assessment period.

#### Experimental Session 1

Upon arrival at the laboratory, participants were seated in a soundproofed testing booth facing a 27″ LCD monitor (60 Hz refresh rate) at a viewing distance of about 65 cm. Prior to the trauma film procedure, participants were subjected to a short reaction time task unrelated to the current research question (Tottenham et al., 2011). Upon completion, electrode setup for physiological stress measures [ECG and EDA; see Toronto Alexithymia Scale (TAS-26; Kupfer et al., 2001) for details] was performed and participants were asked to fill out two standardized questionnaires (State-Trait Anxiety Inventory – State Version; STAI-S; Spielberger, 2010 and Emotional Intensity Scale; EIS; Stone and Nielson, 2001). These served as a baseline measure of mood states (pre-film measurement). Thereafter, baseline physiological parameters were assessed while participants viewed a black screen for a duration of 5 min (pre-film measurement). After measurements were completed, participants received standardized instructions on the trauma film procedure. They were informed that they would be exposed to different film clips via computer screen and headphones, which they should pay attention to while imagining that they were an eyewitness to what was happening. It was emphasized that withdrawal from further study participation was possible at any time. Following these instructions, trauma films were presented and physiological recordings were continued throughout the experimental manipulation (peri-film measurement). After the film had ended, participants were asked to complete the STAI-S (Spielberger, 2010) and the EIS (Stone and Nielson, 2001) again, followed by another 5-min interval of physiological measurements (post-film measurement). Prior to leaving the laboratory, participants received an intrusion diary (to be completed throughout days 0 to 3, starting immediately after the experimental session; see Physiological stress measurements for details) as well as an evening questionnaire, assessing daily stress and arousal levels (to be completed in the evening starting on day 1; see Physiological stress measurements for details). They received oral and written instructions on the characteristics of intrusive memories and how these differ from the experience of conscious memory retrieval.

#### Experimental Session 2

Following three intervening days of ambulatory intrusion assessment, participants returned to the laboratory on day 4 and were seated in a testing booth after handing in their intrusion diary and evening questionnaire. Participants were instructed that they would now perform a memory task (see Memory task for details). They were informed that this task was unrelated to the previous experimental procedures. Subsequent to the memory task, participants were informed that the experiment was over and given the monetary compensation. Further, they were invited to talk about the experiment if they felt the need to do so. Finally, contact details of the experimenter were given to them, which contained an encouragement to get in touch in case they felt any kind of uneasiness or distress related to the experiment.

### Materials and Measures

#### Paradigms

##### Trauma film

The trauma film used in the current study consisted of different film clips taken from a variety of commercially available R-rated movies (e.g., *German Angst*, *I spit on your grave 2*). Participants were informed in the study advertisement, letter of introduction, and informed consent process that the film contained graphic material that could be disturbing, and that they were free to withdraw at any time without any disadvantages. All film clips contained depictions of extreme physical and/or sexual violence. Individual clips were selected based on the results of a pilot study in which an unrelated sample of participants (*N* = 14) rated different preselected film clips with regard to their emotional impact and aversiveness. Clips with the highest mean ratings were compiled to form a 14-min-long film. To prevent order effects, individual film clips were presented counterbalanced across all participants.

##### Memory task

The recognition memory task was adapted from a previous study (Sopp et al., 2017). It comprised an encoding and a retrieval phase. During the encoding phase, negative and neutral pictures were presented on different screen locations (right or left) with the instruction to retain their content as well as their position. During the retrieval phase, participants were instructed to distinguish old and new pictures^1^ and additionally indicate the original screen location, whenever applicable. This procedure enables the assessment of source memory (memory for a specific episodic detail) alongside item memory performance (Yonelinas, 2002).

Pictures were selected from the IAPS (Lang et al., 2005) and supplemented with pictures from the Nencki Affective Picture System (Marchewka et al., 2014). Negative pictures were preselected to be low in valence (*M* = 2.53, *SD* = 0.66) and moderate to highly arousing (*M* = 6.25, *SD* = 0.67), whereas neutral stimuli were chosen to be medium in valence (*M* = 5.5, *SD* = 0.61) and moderate to low in arousal (*M* = 4.01, *SD* = 0.81). In the current version of the task, participants encoded 200 pictures (100 pictures of each stimulus category). These were presented intermixed with 200 new pictures during the retrieval test resulting in a total of 400 test trials. Subsets of old and new images were matched in stimulus properties (valence, arousal, luminance, animacy, displays of faces, and indoor/outdoor scenes) and counterbalanced across participants. A detailed description of the encoding and retrieval procedure can be found in the Supplementary Materials.

Previous studies link trait alexithymia with deficits in emotional item memory performance (Luminet et al., 2006; Vermeulen and Luminet, 2009; Meltzer and Nielson, 2010; Vermeulen et al., 2010; Donges and Suslow, 2015). In order to expand on these findings in the context of analog PTSD symptoms, we focused our analyses on item memory (referred to as recognition memory) performance^2^. To this end, we calculated percentages of correctly identified “old” (hits) and “new” (correct rejections; CRs) pictures separately for each stimulus category (emotional/neutral). These percentages were further aggregated by computing the old/new discrimination PR index (PR = hits – false alarms; Snodgrass and Corwin, 1988). Reaction times (RTs) on individual trials were averaged across hits and CRs.

#### Measures

##### Toronto Alexithymia Scale (TAS-26; *Kupfer et al., 2001*)

The Toronto Alexithymia Scale (TAS-26) is an adapted German version of the TAS-26 (Taylor et al., 1985), which measures trait alexithymia on a self-report basis. The questionnaire consists of 26 items, which are rated on a 5-point-Likert scale ranging from 1 (“strongly disagree”) to 5 (“strongly agree”). Individual ratings of 18 items can be aggregated to form three subscale scores (“Difficulties identifying feelings” – DIF; “Difficulties describing feelings” – DDF; “Externally oriented thinking” – EOT) as well as a global sum score which ranges from 18 to 90. Internal consistency and reliability of the DDF and EOT subscales have been found to be satisfactory. The sum score and the DIF subscale have further been demonstrated to exhibit good psychometric properties (Kupfer et al., 2001).

##### Physiological stress measurements

Physiological recordings were collected using an ActiveTwo amplifier (BioSemi, Amsterdam, Netherlands) at a sampling rate of 512 Hz. For heart rate (HR) measurement, a standard lead-II electrocardiogram (ECG) with two Ag/AgCl electrodes was used to collect a raw ECG signal. R-waves were identified automatically by ANSLAB (Wilhelm and Peyk, 2005) and edited manually for artifacts, false positives or non-recognized R-waves and transformed into instantaneous HRs. To measure skin conductance levels (SCL), two Ag/AgCl electrodes filled with isotonic electrode gel were attached to the proximal part of the palm of the participant’s non-dominant hand (with an alternating current of 1 mA synchronized with the sampling frequency passed between the electrodes). The recorded signal was decimated to 25 Hz, edited manually for artifacts and smoothed using a 1 Hz low-pass filter. As measures of primary interest, means of HR and SCL were calculated prior to (pre), during (peri) and following (post) film presentation.

##### Ambulatory assessment of intrusions and daily distress

Participants were asked to complete an intrusion diary starting after the first laboratory session (day 0) and for three consecutive days (days 1 to 3). Participants were instructed to carry the intrusion diary with them during the whole assessment period and document every intrusive memory immediately after its occurrence. Intrusions were defined as recurrent, sudden, spontaneous and non-initiated memories of film scenes that are very vivid and consist of pictures, sounds, thoughts, words or sentences, feelings or combinations of those. Participants were carefully instructed that intrusions do not include reflective and conscious thinking or deliberate thoughts about the film scenes, which were not to be recorded in the diary. For each intrusion, participants provided a brief description of its content as well as the exact time and cause (if identifiable) of its occurrence. In addition, they were asked to rate distress (10-point scale from “not at all” to “extremely”), valence (5-point scale from “unhappy” to “happy”), and arousal (5-point scale from “unaroused” to “aroused”) experienced during the intrusion. Based on previous findings (Streb et al., 2016, 2017; Holz et al., 2017; Rattel et al., 2018), we focused the current analyses on intrusion distress ratings.

In a separate section of the diary (evening questionnaire), participants were asked to indicate their daily stress and arousal levels. Starting on the evening of day 1, daily stress and arousal levels were rated on a scale from 0 (“none”) to 10 (“very high”) for three consecutive days. Instructions did not include any specific reference to the trauma film.

### Statistical Analyses

Participants with higher (HTA) and LTA levels were determined based on a median split (Md = 39) of TAS-26 sumscores (see e.g., Vermeulen et al., 2010). Differences in trauma film responses, intrusion frequency, and episodic memory performance were analyzed by means of separate analysis of variance (ANOVAs) with group as independent variable (HTA/LTA). Due to the limited overall amount of intrusions reported during the ambulatory assessment period, distress ratings were averaged across all intrusions for subsequent analyses^3^.

In order to examine alexithymia subdomains, correlational analyses were performed between variables of interest and TAS-26 subscale scores. Due to the restricted sample size in certain correlational analyses, non-parametric (Kendall’s τ) correlation coefficients are reported throughout. The alpha level for all analyses was set to 0.05 and significant main or interaction effects of ANOVAs were further explored using *t*-tests. When the sphericity assumption was violated, analyses included Greenhouse-Geisser corrections for non-sphericity with corrected *p*-values and uncorrected degrees of freedom. Due to missing values, degrees of freedom varied across analyses. All statistical analyses were calculated using IBM SPSS Statistics 22 (IBM Corp., Armonk, NY, United States).

## Results

### Pre- and Peritraumatic Measures

#### Descriptive Statistics of TAS-26 Scores

The mean TAS-26 score across all participants was 40.15 (*SD* = 8.48). Mean subscale scores were similar for all three alexithymia sub dimensions: “Difficulties identifying feelings” (DIF; *M* = 13.15, *SD* = 4.14), “Difficulties describing feelings” (DDF; *M* = 13.11, *SD* = 4.15), and “Externally oriented thinking” (EOT; *M* = 13.88, *SD* = 3.02). Characteristics of HTA (*N* = 13) and LTA (*N* = 13) groups are reported in Table 1. Groups differed on all TAS-26 subscales and were comparable in age and gender distribution.

**Table 1:** Demographic characteristics and alexithymia scores in the HTA and LTA groups.

	LTA group *N* = 13	HTA group *N* = 13
Age	23.00 (3.85)	20.70 (2.69)
Gender	2 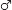 / 11 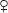	2 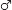 / 11 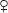
TAS-26 sum score	33.08 (4.27)	47.23 (4.81)^∗∗^
DIF score	10.08 (2.60)	16.23 (2.92)^∗∗^
DDF score	10.31 (3.04)	15.92 (3.10)^∗∗^
EOT score	12.69 (3.04)	15.08 (2.60)^∗^

*TAS-26, Toronto Alexithymia Questionnaire; DIF, Difficulties in Identifying feelings; DDF, Difficulties in describing feelings; EOT, Externally oriented thinking; HTA, Higher trait alexithymia; LTA, Lower trait alexithymia. ^∗∗^Indicates p < 0.001; ^∗^indicates p < 0.05. Standard deviations are given in parentheses.*

#### Trauma Film Paradigm

Subjective and physiological stress levels were analyzed to confirm the validity of the trauma film paradigm and examine differences between HTA and LTA participants.

##### Subjective stress measures

An ANOVA of state-anxiety levels (STAI-S) including the factors Time (pre/post-film) and Group (HTA/LTA) revealed a significant main effect of Time (*F*_1,24_ = 44.46, *p* < 0.001, ${\text{η}}_{\text{p}}^{2}$ = 0.65). Thus, state-anxiety levels increased significantly from pre- to post-film measurement in HTA and LTA participants (Group: *F*_1,24_ = 2.80, *p* = 0.108, ${\text{η}}_{\text{p}}^{2}$ = 0.10; Group × Time: *F*_1,24_ = 0.43, *p* = 0.517, ${\text{η}}_{\text{p}}^{2}$ = 0.02). Analyses of emotional intensity ratings (EIS) similarly revealed a significant effect of Time (*F*_1,24_ = 29.45, *p* < 0.001, ${\text{η}}_{\text{p}}^{2}$ = 0.55) indicating an enhanced emotional intensity at post-film measurement in the absence of any group-related effects (Group: *F*_1,24_ = 0.92, *p* = 0.348, ${\text{η}}_{\text{p}}^{2}$ = 0.04; Group × Time: *F*_1,24_ = 0.01, *p* = 0.978, ${\text{η}}_{\text{p}}^{2}$ < 0.01).

##### Physiological stress measures

**FIGURE 2:**
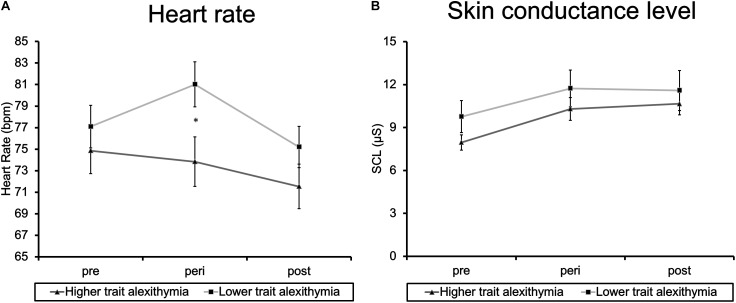
Group means of **(A)** heart rate and **(B)** skin conductance level at pre-, peri-, and post-film assessment. Error bars represent standard error of the mean, ^∗^indicates *p* < 0.05.

Physiological measures (HR and SCL) were subjected to separate ANOVAs including the factors Time (pre/peri/post-film) and Group (HTA/ LTA). Results of HR analyses demonstrate a significant main effect of Time (*F*_2,40_ = 9.91, *p* = 0.001; ${\text{η}}_{\text{p}}^{2}$ = 0.33) and a significant interaction of Time and Group (*F*_2,40_ = 3.80, *p* = 0.046, ${\text{η}}_{\text{p}}^{2}$ = 0.16) in the absence of a main effect of Group (*F*_1,20_ = 0.57, *p* = 0.461, ${\text{η}}_{\text{p}}^{2}$ = 0.03). *Post hoc t*-tests revealed a significant increase of HR in the LTA group from pre-film to peri-film assessment (*t*_11_ = 2.46, *p* = 0.032) which was not evident in the HTA group (*t*_9_ = 0.85, *p* = 0.418; see Figure 2A). The amount of increase also differed significantly between LTA (*M* = 3.92, *SD* = 5.51) and HTA (*M* = -1.02, *SD* = 3.80) participants (*t*_20_ = 2.40, *p* = 0.027). From peri-film to post-film assessment, LTA participants exhibited a significant decline in HR (*t*_11_ = 3.42, *p* = 0.006). This decline was not significant in HTA participants (*t*_9_ = 1.97, *p* = 0.080). However, differences in post-film decrease did not reach the level of significance in between-group comparisons (*t*_20_ = 1.63, *p* = 0.118).

Analyses of SCL revealed a main effect of Time (*F*_2,40_ = 9.67, *p* = 0.003, ${\text{η}}_{\text{p}}^{2}$ = 0.37) in the absence of any further effects (Group: *F*_1,20_ = 0.21, *p* = 0.651, ${\text{η}}_{\text{p}}^{2}$ = 0.10; Group × Time: *F*_2,40_ = 0.29, *p* = 0.656, ${\text{η}}_{\text{p}}^{2}$ = 0.04). A significant rise in SCL was evident from pre-film to peri-film assessment (*t*_21_ = 3.45, *p* = 0.002), which was maintained over time as reflected in a non-significant decline from peri-film to post-film assessment (*t*_21_ = 0.27, *p* = 0.791; see Figure 2B).

In summary, these results confirm the validity of our experimental manipulation. State anxiety, emotional intensity, and physiological stress levels (HR and SCL) were found to increase significantly as a result of trauma film exposure. Interestingly, this increase was not evident in the HR data of HTA participants. HTA and LTA individuals thus exhibited different peritraumatic response patterns. To follow up on this effect, we examined correlations between the level of HR increase (peri-film – pre-film) and TAS-26 subscale scores. Analyses yielded a single significant correlation between HR increase and the TAS-26 DIF subscale (*r_τ_* = -0.31, *p* = 0.047; all other -0.21 > *r_τ_* > -0.29, all other *p* > 0.06), reflecting reduced HR increases in individuals reporting enhanced difficulties in identifying feelings.

### Trait Alexithymia and Analog Symptoms of Traumatic Film Exposure

#### Group Comparison of Ambulatory Intrusion Frequency, and Distress Ratings

**FIGURE 3:**
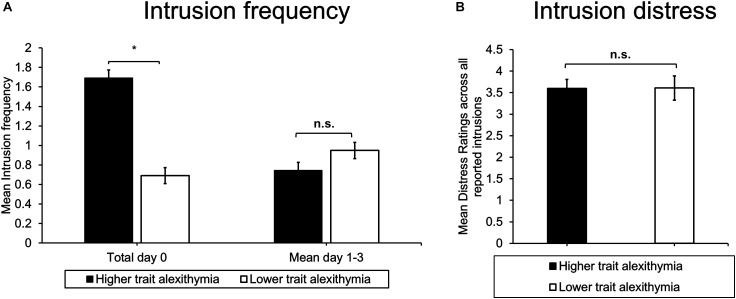
Ambulatory **(A)** intrusion frequency and **(B)** mean intrusion distress ratings for higher and lower trait alexithymia (LTA) subgroups. Error bars represent standard error of the mean, ^∗^indicates *p* < 0.05.

An ANOVA of intrusion frequency including the factors Time (day 0 to 3) and Group (HTA/LTA) revealed a main effect of Time (*F*_3,72_ = 3.90, *p* = 0.023, ${\text{η}}_{\text{p}}^{2}$ = 0.14). As commonly found in trauma film experiments, intrusion frequency declined significantly across time (intrusion frequency day 0 vs day 3: *t*_25_ = 3.36, *p* = 0.002). Moreover, a significant interaction emerged between Group and Time (*F*_3,72_ = 3.93, *p* = 0.023, ${\text{η}}_{\text{p}}^{2}$ = 0.14) in the absence of a main effect of Group (*F*_1,24_ = 0.05, *p* = 0.822, ${\text{η}}_{\text{p}}^{2}$ < 0.01). *Post hoc* tests confirmed a significant between-group difference on day 0 (*t*_24_ = 2.16, *p* = 0.041) indicating that HTA participants reported more intrusions than LTA participants on the day of “traumatic” exposure (see Figure 3A). However, this difference was not maintained across subsequent days of ambulatory intrusion assessment (day 1: *t*_24_ = 0.99, *p* = 0.332; day 2: *t*_24_ = 0.43, *p* = 0.674; day 3: *t*_24_ = 0.53, *p* = 0.604). Analyses of intrusion distress ratings did not reveal a significant between-group difference (*t*_18_ = 0.02, *p* = 0.988; see Figure 3B).

#### Correlational Analyses of Alexithymia, Intrusive Memories and Ratings of the Evening Questionnaire

Correlational analyses between TAS-26 subscales, intrusion frequency (sum score across days 0 to 3), and mean intrusion distress ratings did not yield any significant results (all 0.09 > *r_τ_* > -0.24, all *p* > 0.150). Analyses of mean daily stress and arousal levels revealed a significant correlation between the TAS-26 DDF subscale and daily stress levels (*r_τ_* = 0.29, *p* = 0.048). Difficulties in describing feelings were thus positively associated with general stress levels after exposure to a “traumatic” event. None of the other correlations reached significance (all other 0.09 > *r_τ_* > -0.19, all other *p* > 0.210).

### Trait Alexithymia and Emotional Memory Performance

#### Group Comparison of Accuracy

An ANOVA of memory performance (% correct) including the within-subject factors Emotion (emotional/neutral) and Response type (hits/CRs) and the between-subject factor Group (HTA/LTA) revealed a significant main effect of Emotion (*F*_1,23_ = 14.83, *p* = 0.001, ${\text{η}}_{\text{p}}^{2}$ = 0.39) and Response type (*F*_1,23_ = 61.43, *p* < 0.001, ${\text{η}}_{\text{p}}^{2}$ = 0.73). A significant interaction effect between Emotion and Response type was also evident (*F*_1,23_ = 53.26, *p* < 0.001, ${\text{η}}_{\text{p}}^{2}$ = 0.70). None of the group-related effects reached significance (Group: *F*_1,23_ = 1.54, *p* = 0.227, ${\text{η}}_{\text{p}}^{2}$ = 0.06; Group × Response type: *F*_1,23_ = 3.04, *p* = 0.095, ${\text{η}}_{\text{p}}^{2}$ = 0.12; Group × Emotion: *F*_1,23_ = 0.01, *p* = 0.911, ${\text{η}}_{\text{p}}^{2}$ < 0.01; Group × Response type × Emotion: *F*_1,23_ = 0.09, *p* = 0.773, ${\text{η}}_{\text{p}}^{2}$ < 0.01).

**FIGURE 4:**
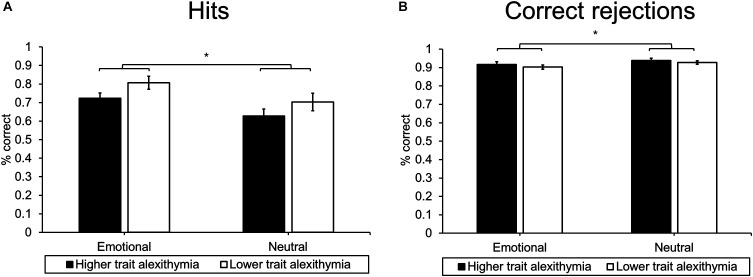
Mean **(A)** hits and **(B)** CRs for higher and LTA subgroups. Error bars represent standard error of the mean, ^∗^indicates *p* < 0.05.

*Post hoc* analyses revealed that participants were more accurate at rejecting new pictures than at recognizing old pictures. In addition, accuracy rates differed for emotional and neutral images in both response categories. Participants exhibited higher hit rates for emotional as compared to neutral images (*t*_24_ = 5.84, *p* < 0.001; see Figure 4A). However, when correctly rejecting new images, participants exhibited higher accuracy rates for neutral as compared to emotional images (*t*_24_ = 4.07, *p* < 0.001; see Figure 4B). This pattern of results mirrors general effects of emotion on episodic memory performance (Maratos et al., 2000; Kensinger, 2009).

In order to account for differences in false alarm rates, we analyzed corrected hit rates (PRs) in a separate ANOVA including the within-subject factor Emotion (emotional/neutral) and the between-subject factor Group (HTA/LTA). The analysis revealed a significant main effect of Emotion (*F*_1,23_ = 14.83, *p* = 0.001, ${\text{η}}_{\text{p}}^{2}$ = 0.39) without any further main or interaction effects (Group: *F*_1,23_ = 1.54, *p* = 0.227, ${\text{η}}_{\text{p}}^{2}$ = 0.06; Group × Emotion: *F*_1,23_ = 0.01, *p* = 0.911, ${\text{η}}_{\text{p}}^{2}$ < 0.01). In line with hit rates, corrected recognition scores were significantly higher for emotional images (*M* = 0.67, *SD* = 0.13) as compared to neutral images (*M* = 0.60, *SD* = 0.16).

#### Group Comparison of Reaction Times

Analyses of RTs (in ms) including the factors Emotion (emotional/neutral), Response type (hits/CRs), and Group (HTA/LTA) yielded significant effects of Emotion (*F*_1,23_ = 39.82, *p* < 0.001, *${\text{η}}_{\text{p}}^{2}$* = 0.63), Response type (*F*_1,23_ = 45.45, *p* < 0.001, ${\text{η}}_{\text{p}}^{2}$ = 0.66) and a significant interaction of Emotion and Response type (*F*_1,23_ = 19.65, *p* < 0.001, ${\text{η}}_{\text{p}}^{2}$ = 0.46). Participants responded significantly faster when accurately rejecting new images (CRs) than when correctly recognizing old images (Hits). In addition, response latencies were significantly enhanced for emotional as compared to neutral images (*t*_24_ = 9.02, *p* < 0.001) when accurately rejecting new items (CRs). No main or interaction effect involving the Group factor emerged from these analyses (Group: *F*_1,23_ = 1.18, ${\text{η}}_{\text{p}}^{2}$ = 0.05, *p* = 0.289; Group × Emotion: *F*_1,23_ = 0.09, *p* = 0.767, ${\text{η}}_{\text{p}}^{2}$ < 0.01; Group × Response types: *F*_1,23_ = 0.78, *p* = 0.388, ${\text{η}}_{\text{p}}^{2}$ = 0.03; Group × Emotion × Response types: *F*_1,23_ = 0.01, *p* = 0.984, ${\text{η}}_{\text{p}}^{2}$ < 0.01).

### Correlational Analyses of TAS-26 Scores and Emotional Memory Performance

**FIGURE 5:**
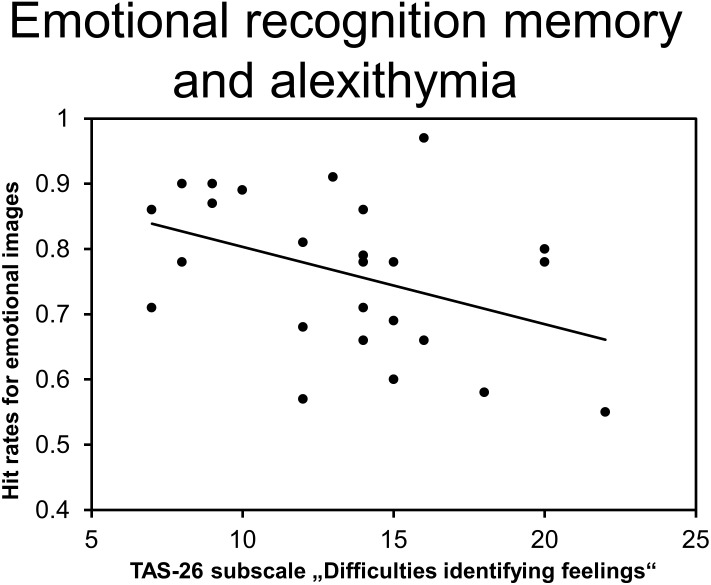
Correlation between the TAS-26 subscale “Difficulties in identifying feelings” and emotional hit rates.

In order to explore whether particular subdomains of trait alexithymia were related to episodic memory performance, we examined non-parametric correlations between behavioral performance (% correct) and RTs (ms) of the recognition memory procedure and TAS-26 subscale scores. Analyses revealed a significant association between DIF subscale scores and hit rates for emotional images (*r_τ_* = -0.29, *p* = 0.050; see Figure 5) but not between DIF scores and hit rates for neutral images (*r_τ_* = -0.16, *p* = 0.268). Trait alexithymia, as reflected in DIF scores, was selectively related to decreased emotional recognition memory. None of the other correlations reached the level of significance (all other 0.22 > *r*_τ_ > -0.24, all other *p* > 0.100).

### Emotional Memory Performance and Analog Symptoms

#### Correlational Analyses of Emotional Memory Performance and Intrusive Memories

To determine whether intrusion frequency was related to episodic memory performance, we calculated non-parametric correlations between intrusion frequency and distress across the ambulatory assessment period and recognition memory performance (accuracy and RTs). No selective correlations for emotional stimuli emerged from these analyses. However, this lack of correlations may be accounted for by low overall intrusion frequency. As such, inter-individual variance in intrusion frequency was most pronounced on the day of traumatic film exposure (day 0) and declined rapidly on the following days (day 1 to 3). To emphasize variance in intrusion frequency on day 0, we computed an index of relative intrusion frequency [relative intrusion frequency = intrusions_day0÷(intrusions_day0+intrusions__day1+intrusions_day2+intrusions_day3)] and subjected this index to exploratory correlational analyses.

#### Exploratory Analyses of Relative Intrusion Frequency on the Initial Day of Film Exposure

**FIGURE 6:**
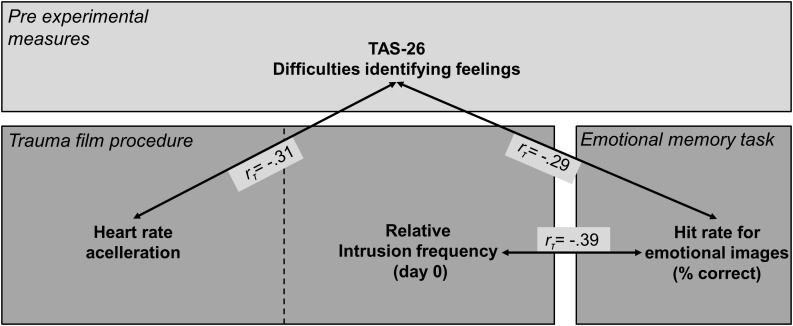
Summary of correlations between DIF subscale scores, variables of the trauma film procedure, and emotional memory performance. Heart rate acceleration refers to the increase of heart rate (bpm) from pre- to peri-film assessment. Relative intrusion frequency refers to the number of intrusions on day 0 divided by the total number of intrusions of the ambulatory assessment period (days 0 to 3).

Exploratory analyses revealed a significant correlation between relative intrusion frequency and emotional hit rates (*r_τ_* = -0.39, *p* = 0.024), which was not evident for neutral hit rates (*r_τ_* = -0.18, *p* = 0.277). Participants with an enhanced relative intrusion frequency on the initial day of “traumatic” exposure thus demonstrated a lower accuracy in recognizing emotional images during the episodic memory task. This finding complements the negative association between DIF scores and emotional item hits (see Figure 6 for illustration). A direct link between DIF scores and relative intrusion frequency was not evident (*r_τ_* = 0.14, *p* = 0.425). Taken together, these correlations suggest that an attenuation of emotional recognition memory was associated with greater pre-experimental difficulties in identifying feelings as well as an enhanced relative intrusion frequency on the day of trauma film exposure.

## Discussion

The current study aimed to provide first insights on the association between trait alexithymia and intrusive re-experiencing. This association was examined in a controlled experimental approach using the trauma film paradigm. In addition, the study design included an assessment of episodic memory performance for neutral and emotional stimulus material. This combined study procedure allowed us to test whether participants with higher alexithymia levels report enhanced intrusive re-experiencing. Moreover, we were able to examine emotional episodic memory performance in these same individuals and explore whether memory performance is related to experimentally induced intrusion frequency.

### Trait Alexithymia in the Context of Traumatic Exposure

#### Effects of Trait Alexithymia on Emotional Responding During the Trauma Film

Analyses of subjective and physiological responses to the trauma film suggest that high alexithymic individuals may exhibit a distinct peritraumatic response pattern. Mood states changed significantly from pre- to post-film assessment in both HTA and LTA participants. However, HTA participants did not show a significant increase in HR from pre- to peri-film assessment. This finding is in line with previous accounts of diminished HR acceleration during affective processing in high alexithymic individuals (Wehmer et al., 1995; Linden et al., 1996; Friedlander et al., 1997; Roedema and Simons, 2001; Torrado et al., 2015; Kleiman et al., 2016; but see Stone and Nielson, 2001; Eastabrook et al., 2013; Hua et al., 2014). In the current study, mean HR changes in HTA participants exhibited a non-significant trend toward HR deceleration. This is particularly noteworthy as HR acceleration during the trauma film has been shown to correlate negatively with subsequent intrusion frequency (Holmes et al., 2004). It has been proposed that HR deceleration in response to traumatic film clips may reflect fear bradycardia responses (Arnaudova and Hagenaars, 2017), which may be accompanied by dissociation. States of dissociation have further been associated with disruptions of peritraumatic encoding processes (Holmes et al., 2004), which could result in enhanced intrusive re-experiencing (see e.g., Sündermann et al., 2013). Although the current study design did not include state- or trait-related measures of dissociation, previous studies suggest that alexithymia is positively correlated with dissociative symptoms (see e.g., Craparo et al., 2014; Terock et al., 2016). Based on these correlations and our preliminary findings, future trauma film studies should examine associations between alexithymia, dissociation, and HR deceleration in greater depth.

#### Effects of Trait Alexithymia on Ambulatory Analog Symptoms

The main objective of the current study was to examine the impact of alexithymia on intrusive re-experiencing. In extension of a previous account linking alexithymia with enhanced implicit retrieval of trauma-related material (Minshew and D’Andrea, 2015), our results reveal an enhanced intrusion frequency in HTA participants on the day of trauma film presentation. This initial enhancement did not persist across the entire assessment period. However, it is important to consider the rapid decline of intrusion frequency across both groups (day 1: *M* = 1.23, *SD* = 1.58; day 2: *M* = 0.81, *SD* = 1.36; day 3: *M* = 0.50; *SD* = 1.10), which likely precluded detecting any between-group differences on the succeeding days of ambulatory assessment. Transient and overall moderate analog symptoms are a frequent disadvantage of experimental approaches, as the rigorous exclusion process prior to study admission results in the selection of a generally healthy group of participants with high levels of psychosocial functioning (for a similar line of argument see Das et al., 2016; Holz et al., 2017). Hence, our findings reveal significant differences in intrusion frequency during the time period most sensitive to detect these differences in a healthy group of participants.

As such, the current results provide preliminary indications that alexithymia may constitute a risk factor for the development of intrusive memories. Although re-experiencing symptoms have been found to be a strong predictor of PTSD (Michael et al., 2005b), it is important to stress that the scope of our results is limited to intrusion development in healthy participants. Thus, future studies are required to confirm our results in clinical samples. Moreover, longitudinal studies need to examine whether effects of alexithymia on intrusive re-experiencing are associated with a higher risk for PTSD. In this regard, potential interactions between alexithymia, initial intrusion frequency, and other PTSD symptom domains should be considered. For instance, initial intrusion frequency has been found to predict subsequent ruminative thinking, which may in turn contribute to the persistence of re-experiencing symptoms (Holz et al., 2017). To address these interactions, future studies should incorporate comprehensive measurements of different PTSD symptoms (e.g., ruminative thinking).

### Associations of Alexithymia, Emotional Memory Performance and Intrusion Frequency

The second objective of the current study was to investigate whether deficits in emotional memory processes are associated with enhanced intrusive re-experiencing in HTA participants. In line with the literature (Maratos et al., 2000; Kensinger, 2009), participants responded with enhanced hit and false alarm rates to emotional stimuli, resulting in overall enhanced emotional recognition performance. Further analyses revealed that the enhancement of hit rates for emotional items was inversely correlated with DIF subscale scores. This finding is in agreement with previous research demonstrating selective associations between emotional memory performance and the DIF scale (Suslow et al., 2003; Vermeulen and Luminet, 2009) and provides further evidence of alexithymia-linked deficits in emotional recognition memory (Luminet et al., 2006; Vermeulen et al., 2010; Donges and Suslow, 2015). However, in contrast to our hypothesis, analyses did not reveal a significant correlation between emotional hit rates and overall intrusion frequency. This lack of association is not surprising, given that intrusion frequency approached floor levels during the second half of the assessment period (see Effects of Trait Alexithymia on Ambulatory Analog Symptoms). Exploratory analyses, which were focused on the time period of greatest between-subject variability, support our assumption: Relative intrusion frequency on the day of “traumatic” exposure was negatively correlated with hit rates for emotional items. Although this finding has to be interpreted with caution, it provides tentative support for the relevance of emotional memory processes in the association between trait alexithymia and intrusive re-experiencing.

By establishing a first link between clinical findings of heightened alexithymia levels in PTSD patients (Yehuda et al., 1997; Kupchik et al., 2007; Evren et al., 2010; Reeves et al., 2012) and experimental evidence of deficient emotional memory processing in alexithymic individuals (Taylor and Bagby, 2004; Luminet et al., 2006; Vermeulen and Luminet, 2009; Meltzer and Nielson, 2010; Vermeulen et al., 2010; Donges and Suslow, 2015), our study highlights an important perspective for future research. As such, the present results set the stage for comprehensive analog studies investigating implicit and explicit memory for “traumatic” material in alexithymic individuals. To ascertain whether effects are selectively evident for trauma-related material, future studies should include a neutral film and measure intrusions and memory performance for each film type (see e.g., Streb et al., 2016; Sopp et al., 2019).

### Limitations and Outlook

Although revealing several interesting indications for further study, our results bear certain limitations. First of all, we used the trauma film paradigm to examine analog PTSD symptoms. The trauma film paradigm is an established method, which has been found useful in studying PTSD symptom development in contexts requiring the systematic assessment of pretraumatic factors (Holmes and Bourne, 2008; James et al., 2016). Nevertheless, as noted above, this approach limits the validity of our findings with regard to symptom development following real-life traumatic events. Beyond this general limitation, it is important to consider the restricted range in trait alexithymia in the current study. Hence, our sample did not exhibit the full range of TAS-26 scores, which may have partially arisen from the comprehensive exclusion procedure prior to study admission (see Effects of Trait Alexithymia on Ambulatory Analog Symptoms). Given that alexithymia is conceptualized as a dimensional construct, we divided our sample into higher and lower scoring subsamples. Nevertheless, it is important to note that the group median was substantially lower than the suggested cut-off score (≥54; Kupfer et al., 2001). Although one would expect more robust effects in a sample with high scoring individuals, future studies are required to re-examine the found effects in low, medium, and high scoring individuals (see e.g., Linden et al., 1996). Moreover, future studies should consider classifying different levels of alexithymia using multiple methods (e.g., self and observer ratings). In line with our approach, previous studies mostly rely on self-report questionnaires to classify high- and low scoring groups (see e.g., Luminet et al., 2006; Meltzer and Nielson, 2010; Vermeulen et al., 2010). However, the validity of this classification could be restricted as alexithymic individuals may lack insight into their emotional processing deficits (see e.g., Rosenberg et al., 2016). Finally, the relatively small sample size and our non-probabilistic sampling strategy may limit the generalizability of our findings. Thus, our results should be confirmed in larger, randomly drawn samples.

Despite its preliminary character, the current study may serve as a starting point for further research into the role of trait alexithymia in intrusion development. In the long run, this line of research could yield important implications for the prevention and treatment of PTSD. Trait alexithymia may thus be an important vulnerability factor to be considered in at-risk populations (e.g., in occupational settings). Moreover, taking account of this factor may help to improve psychotherapeutic treatment outcomes (Quirin et al., 2014). Brady et al. (2015) found that patients with alexithymia-like trait characteristics, as evaluated in the initial therapy session, showed restricted symptom remission across subsequent trauma-focused cognitive therapy (see also O’Brien et al., 2008). Further research indicates that high alexithymic individuals benefit less from psychotherapy in general and that these effects may be specifically related to their deficits in emotion processing and expression (Ogrodniczuk et al., 2011). These findings illustrate a potential to enhance treatment outcomes by assessing alexithymia levels prior to treatment and targeting associated deficits during subsequent sessions. Although specific interventions addressing alexithymia have not been developed to date, several lines of treatment (e.g., emotional processing training, pharmacological adjuncts, and affect labeling training) may prove to be effective in the future (Samur et al., 2013).

## Data Availability Statement

The raw data supporting the conclusions of this manuscript will be made available by the authors, without undue reservation, to any qualified researcher.

## Author Contributions

MS, AB, and TM conceptualized the study design. MS and AB were responsible for data acquisition and analyses. MS drafted the first version of the manuscript. TM provided critical revisions. All authors read and approved the submitted version.

## Conflict of Interest Statement

The authors declare that the research was conducted in the absence of any commercial or financial relationships that could be construed as a potential conflict of interest.

## Footnotes

1 The retrieval test entailed a Remember-Know-New procedure (Rajaram, 1993). Results are reported collapsed across R- and K- responses as separate analyses yielded identical results.

2 Analyses focusing on associations between trait alexithymia and source memory performance will be reported elsewhere. Source memory performance was not correlated with any of the trauma film variables.

3 Mean distress ratings were determined by summation of distress ratings of all intrusions (day 0 to 3) and division by the total number of intrusions (day 0 to 3).
