# Supplementary material for: The Prospective Influence of Trait Alexithymia on Intrusive Memories: What Is the Role of Emotional Recognition Memory?
Source: Front Psychol. 2019 Jan 8;9:2642. doi: 10.3389/fpsyg.2018.02642 (PMC6331440; doi:10.3389/fpsyg.2018.02642)
Supplement: Supplementary file 1 [file Table_1.DOCX]

Supplementary Material: Procedures of the Episodic Memory Task

THE PROSPECTIVE INFLUENCE OF TRAIT ALEXITHYMIA ON PTSD SYMPTOMS: WHAT IS THE ROLE OF EMOTIONAL RECOGNITION MEMORY?

**M. Roxanne Sopp^*^, Alexandra H. Brueckner, & Tanja Michael**

*** Correspondence:** Marie Roxanne Sopp: [roxanne.sopp@uni-saarland.de](mailto:roxanne.sopp@uni-saarland.de)

**1. Stimulus Material**

Neutral and negative images were selected from the International Affective Picture System (Lang, Bradley, & Cuthbert, 2005) and supplemented with pictures from the Nencki Affective Picture System (Marchewka, Żurawski, Jednoróg, & Grabowska, 2014). To ascertain the applicability of the norms in the current student population, a prerating of the pictures with an unrelated sample of participants (N= 10) was conducted. Neutral and negative stimuli were closely matched in luminance, animacy, the occurrence of faces and indoor/outdoor displays. The final amount of 200 negative and 200 neutral items was split into 2 parallel sets equal in valence, arousal and the aforementioned visual stimulus properties, which served as old and new items during the test phase respectively and were counterbalanced across participants. To exclude the potential occurrence of primacy and recency effects and to allow adaption to task procedures 3 neutral and 3 negative pictures were presented prior to and following the target picture set. The order of stimulus presentation was pseudo-randomized with no more than two stimuli of the same valence category occurring in succession.

**2. Encoding phase**

During encoding participants saw an initial fixation cross-hair (500ms) after which the stimulus was presented for 2000 ms. The screen location was varied by presenting the stimulus on one of four different screen positions (right and left, as well as near or far from the screen center). After stimulus presentation, the participant’s judgment of the encoding task (“Is the stimulus perceived as being positioned near or far from the screen center?”) was ascertained (2000 ms) with the response keys counterbalanced across participants. This encoding procedure was chosen in order to ensure interactive encoding of the source feature (screen location) and the respective item. Encoding was intentional with participants being instructed to memorize the items and their respective screen locations (right/left) for a subsequent test phase.

**3. Retrieval phase**

The retrieval test was administered 5 minutes after encoding was completed. Participants received standardized instructions regarding the test procedure, which included a remember-know-new decision (Rajaram, 1993). Participants were instructed to determine whether each of the successively presented pictures had previously occurred in the test phase (“Old”) or not (“New”). Whenever a picture was identified as old, they were asked to indicate whether they were able to retrieve specific details of its previous presentation (“Remember”) or not (“Know”).

Each test trial began with the presentation of an initial fixation cross-hair (500ms) which was followed by stimulus presentation (1500 ms) and an additional response window of 2500 ms at stimulus offset (blank screen). Participants were instructed to respond as fast and as accurate as possible with the onset of stimulus presentation by providing a Remember(R)/Know(K)/New(N) response. Response key assignment was again counterbalanced across participants. For items that were indicated to be old (R- or K-response) an additional response window appeared (4000 ms) asking participants to retrieve the initial screen location of the picture (right/left/unknown).

**References**

Lang, P.J., Bradley, M.M., & Cuthbert, B.N. (2005). *International Affective Picture System (IAPS): Digitized Photographs, Instruction Manual and Affective Ratings.* Technical Report A-6, University of Florida, Gainesville, FL.

Marchewka, A., Żurawski, Ł., Jednoróg, K., & Grabowska, A. (2014). The Nencki Affective Picture System (NAPS): Introduction to a novel, standardized, wide-range, high-quality, realistic picture database. *Behavior Research Methods*, *46*(2), 596-610.

Rajaram, S. (1993). Remembering and knowing: Two means of access to the personal past. *Memory & Cognition, 21*(1), 89-102.
